# Does mental health staffing level affect antipsychotic prescribing? Analysis of Italian national statistics

**DOI:** 10.1371/journal.pone.0193216

**Published:** 2018-02-21

**Authors:** Fabrizio Starace, Francesco Mungai, Corrado Barbui

**Affiliations:** 1 Department of Mental Health and Drug Abuse, AUSL Modena, Modena, Italy; 2 WHO Collaborating Centre for Research and Training in Mental Health and Service Evaluation, Department of Neuroscience, Biomedicine and Movement Sciences, Section of Psychiatry, University of Verona, Verona, Italy; RTI International Metals Inc, UNITED STATES

## Abstract

**Introduction:**

In mental healthcare, one area of major concern identified by health information systems is variability in antipsychotic prescribing. While most studies have investigated patient- and prescriber-related factors as possible reasons for such variability, no studies have investigated facility-level characteristics. The present study ascertained whether staffing level is associated with antipsychotic prescribing in community mental healthcare.

**Methods:**

A cross-sectional analysis of data extracted from the Italian national mental health information system was carried out. For each Italian region, it collects data on the availability and use of mental health facilities. The rate of individuals exposed to antipsychotic drugs was tested for evidence of association with the rate of mental health staff availability by means of univariate and multivariate analyses.

**Results:**

In Italy there were on average nearly 60 mental health professionals per 100,000 inhabitants, with wide regional variations (range 21 to 100). The average rate of individuals prescribed antipsychotic drugs was 2.33%, with wide regional variations (1.04% to 4.01%). Univariate analysis showed that the rate of individuals prescribed antipsychotic drugs was inversely associated with the rate of mental health professionals available in Italian regions (Kendall's tau -0.438, p = 0.006), with lower rates of antipsychotic prescriptions in regions with higher rates of mental health professionals. After adjustment for possible confounders, the total availability of mental health professionals was still inversely associated with the rate of individuals exposed to antipsychotic drugs.

**Discussion:**

The evidence that staffing level was inversely associated with antipsychotic prescribing indicates that any actions aimed at decreasing variability in antipsychotic prescribing need to take into account aspects related to the organization of the mental health system.

## Introduction

Reliable and timely health information is the foundation for effective health services management and public health action [1]. In the area of mental health strengthening information systems is one of the ten recommendations issued by the World Health Organization (WHO) to make a difference in mental healthcare [2]. When their potential is fully harnessed, health information systems can generate data to assess and monitor the structure, processes, and outcomes of care [3].

In mental healthcare, one area of major concern identified by health information systems is the appropriate use of antipsychotic (AP) medications [4]. High variability in AP use in community mental healthcare has often been documented, with concerns regarding the appropriateness of therapy, including misuse, underuse and overuse [5]. Interestingly, while most studies have investigated patient- and prescriber-related factors as possible reasons for variability in AP use, almost no studies have investigated facility-level characteristics.

One facility characteristic that may be associated with AP use is staffing levels, as already documented in nursing homes [6]. In these hospital-based settings studies have demonstrated an inverse relationship between staffing levels and AP use, that is, as staffing levels decrease AP drug use increases [6,7]. However, in community mental healthcare there are no data investigating this association. Against this background, given the availability of a recently implemented Italian national mental health information system, this study investigated whether staffing level is associated with AP prescribing in community mental healthcare.

## Methods

### Study setting

The present study was conducted in Italy, a European country located in the heart of the Mediterranean sea. With 60 million inhabitants, it is the fourth most populous European member state. The Italian state runs a universal public healthcare system since 1978. Within this healthcare system, individuals with mental health conditions receive psychiatric care within a specific regional mental health system. Each Italian region set up its own mental health system, and each mental health system is composed by a network of community-based psychiatric services. Each community-based psychiatric service provides care to all residents of a well-defined catchment area. Therefore, from an epidemiological point of view, each region represents a single evaluation unit that is relatively homogeneous in terms of policies, resources, and service delivery. Information on drug treatment is stored by regional administrative databases which include all community (i.e. outside hospitals) prescriptions reimbursed by the National Health System (NHS) for the population living in each region. Therefore, general practitioner prescriptions, ambulatory prescriptions delivered by specialists (psychiatrists, neurologists, others) and prescriptions delivered in private care are included in the database if reimbursed by the NHS. In Italy, typical and atypical AP drugs are fully reimbursed by the NHS on the basis of a favourable cost-effectiveness profile. AP drugs can be prescribed by doctors only, while nurses and other professionals are not allowed to issue prescriptions. Since 1978 the Italian state runs a universal health care system based on public hospitals and private ones that receive public reimbursement for their activities. In both types of facilities, health care is provided completely free of charge to all citizens and residents, regardless of their income.

### Data source

The data used in this study were extracted from the recently implemented Italian national mental health information system [8]. This information system, based on data provided by each Italian region to the Ministry of Health, collects data on the availability and use of mental health facilities. Based on data collected in 2015, the Italian Ministry of health published a first national report on mental health in 2016, and made all data, aggregated at a regional level, publicly available. For the purposes of this analysis, the following information was extracted for each Italian region: total mental health staff (rate per 100,000 inhabitants); psychiatrists (rate per 100,000 inhabitants); mental health nurses (rate per 100,000 inhabitants); psychiatric beds (rate per 100,000 inhabitants); treated prevalence of any mental disorders (number of individuals with at least one contact with psychiatric services during 2015 per 100,000 inhabitants); treated incidence of any mental disorders (number of individuals with a first ever contact with psychiatric services during 2015 per 100,000 inhabitants); treated prevalence of bipolar disorder (number of individuals with a diagnosis of bipolar disorder with at least one contact with psychiatric services during 2015 per 100,000 inhabitants); treated prevalence of schizophrenia and related psychotic disorders (number of individuals with a diagnosis of schizophrenia and related psychotic disorders with at least one contact with psychiatric services during 2015 per 100,000 inhabitants); treated incidence of bipolar disorder (number of individuals with a diagnosis of bipolar disorder with first ever contact with psychiatric services during 2015 per 100,000 inhabitants); treated incidence of schizophrenia and related psychotic disorders (number of individuals with a diagnosis of schizophrenia and related psychotic disorders with a first ever contact with psychiatric services during 2015 per 100,000 inhabitants); psychiatric hospital admissions (rate per 100,000 inhabitants); AP prescribing (number of individuals receiving at least one AP prescription during 2015 per 1,000 inhabitants). AP drugs were identified as medicines belonging to the N05A (with the exception of lithium) category of the Anatomical Therapeutic Chemical Classification System. From the Central Institute of Statistics (ISTAT) we collected additional information on employment (rate per 100 inhabitants) and poverty (number of individuals living below the poverty line per 100 inhabitants) [9].

### Data analysis

As first analytical step, the rate of individuals exposed to AP drugs was tested for evidence of association with the rate of mental health staff (total, nurses, psychiatrists, psychologists, educators/other staff) using Kendall’s rank correlation test. As second analytical step, the rate of individuals exposed to AP drugs was tested for evidence of association with the rate of mental health staff (total, nurses only, psychiatrists only) by means of two linear regression models. In the first model, we adjusted for the following confounding variables: psychiatric beds; treated prevalence of mental disorders; treated incidence of mental disorders, psychiatric hospital admissions; poverty index; employment rate. In the second model, we used the same variables but we replaced the treated prevalence and incidence of mental disorders with the following variables: treated prevalence of schizophrenia; treated prevalence of bipolar disorder; treated incidence of schizophrenia; treated incidence of bipolar disorder. A nonparametric bootstrap method of statistical accuracy was used, assuming that the observed distribution of the present sample was a good estimate of the true population distribution [10]

## Results

In Italy in 2015 there were on average nearly 60 mental health professionals per 100,000 inhabitants, with wide regional variations (range 21 to 100) (Table 1).

**Table 1:** Distribution of mental health staff, psychiatric beds, treated incidence and treated prevalence of mental disorders, poverty index, employment rate and rate of individuals treated with antipsychotics by Italian regions.

Name of Italian region	Rate per 100,000											Rate per 100		Rate per 1,000
	Mental health staff	Psychia-trists	Mental health nurses	Psychiatric beds	Treated prevalence	Treated incidence	Treated prevalen-ce of bipolar disorder	Treated prevalen-ce of schizo-phrenia	Treated incidence of bipolar disorder	Treated incidence of schizo-phrenia	Psychiatric hospital admissions	Poverty index	Employment	Individuals prescribed antipsycho-tic drugs
PIEMONTE	49.27	5.82	24.97	10.98	1637.78	760.43	125.13	274.11	25.37	40.64	457.31	8.9	63.66	31.6
VALLE D'AOSTA	109.29	0.93	33.62	14.01	NA	NA	NA	NA	NA	NA	380.18	10.5	66.18	12.7
LOMBARDIA	60.58	6.43	26.97	9.57	1766.46	475.42	114.99	360.07	16.13	59.51	337.34	8.2	65.13	27.9
BOLZANO	94.60	11.73	34.96	15.08	NA	NA	NA	NA	NA	NA	666.78	5.2	71.37	17.4
TRENTO	78.14	11.35	24.07	10.67	1659.57	602.86	256.22	265.08	70.19	34.07	299.61	7.1	66.08	16.0
VENETO	57.12	7.39	26.56	21.68	1434.03	715.92	122.02	314.19	21.41	38.56	394.12	7.1	63.61	13.2
FRIULI VENEZIA GIULIA	58.50	9.87	26.95	3.45	1165.18	1202.30	45.65	212.93	46.23	216.19	166.41	13.5	63.65	15.7
LIGURIA	83.57	13.57	36.24	11.88	1753.30	1373.22	64.86	154.45	12.03	46.44	541.51	12.5	62.38	18.1
EMILIA ROMAGNA	86.45	13.11	41.33	11.93	2058.16	927.03	164.28	417.50	33.71	50.24	417.64	6.4	66.69	10.4
TOSCANA	60.03	7.58	28.66	10.85	1104.90	414.95	96.60	130.30	19.47	11.35	285.18	6.7	64.81	25.5
UMBRIA	33.60	7.67	20.50	4.89	1648.94	641.98	214.30	307.16	58.20	49.34	202.52	13.6	63.10	14.8
MARCHE	46.91	3.52	26.21	12.57	1589.36	441.95	165.28	417.34	19.08	23.22	377.48	12.0	62.14	25.7
LAZIO	44.96	4.16	22.77	7.25	1385.96	887.58	128.64	287.48	55.42	97.33	214.52	10.3	58.97	28.0
ABRUZZO	35.37	6.84	14.31	8.88	1424.06	695.54	88.08	224.97	27.91	42.84	303.19	12.5	54.50	40.1
MOLISE	20.58	6.73	11.60	8.60	1651.04	738.33	146.31	313.22	28.06	46.77	304.61	22.7	49.38	33.2
CAMPANIA	52.47	5.15	26.15	10.75	1393.91	582.59	92.09	230.55	20.68	41.32	216.20	19.9	39.57	20.2
PUGLIA	44.40	6.25	18.01	6.61	1675.75	838.05	152.18	405.47	40.83	83.91	227.51	21.9	43.29	25.2
BASILICATA	37.41	NA	17.06	6.99	1077.28	510.06	75.03	161.38	13.77	21.38	211.96	24.7	49.19	27.8
CALABRIA	48.95	5.65	17.75	5.59	1613.41	1534.53	52.42	177.09	28.82	103.81	204.88	33.1	38.91	28.6
SICILIA	70.48	10.39	29.27	12.06	1863.32	1016.43	113.54	436.68	37.38	117.23	342.84	30.1	39.97	20.3
SARDEGNA	57.77	14.54	25.30	5.06	NA	NA	NA	NA	NA	NA	329.01	16.8	50.14	26.7

On average, there were 10 psychiatric beds per 100,000 inhabitants (range 5 to 21). The treated prevalence of schizophrenia was 0.31% (range 0.13% to 0.44%) and the treated prevalence of bipolar disorder was 0.12% (range 0.05% to 0.25%), while the treated prevalence of any psychiatric disorder was 1.59% (1.07 to 2.05) The average rate of individuals prescribed AP drugs was 2.33%, with wide regional variations (1.04% to 4.01%) (Table 1).

**Fig 1:**
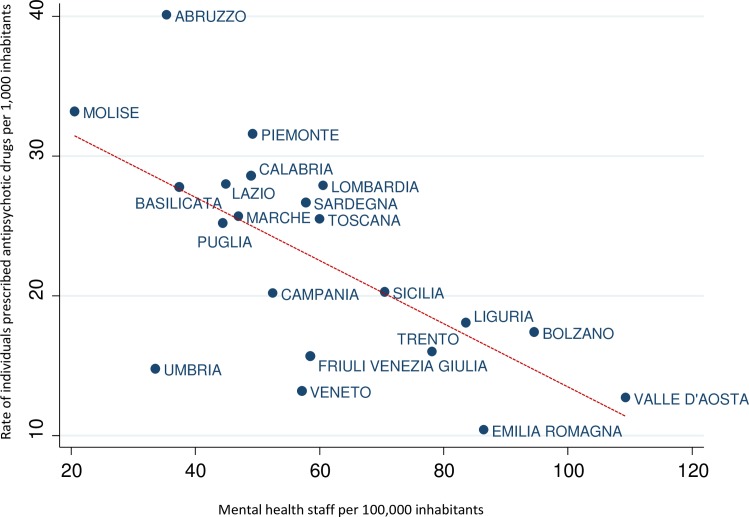
Relationship between staff availability per 100,000 inhabitants and rate of individuals prescribed antipsychotic drugs per 1,000 inhabitants in Italian regions.

Univariate analysis showed that the rate of individuals prescribed AP drugs was inversely associated with the rate of mental health professionals available in Italian regions (Kendall's tau -0.438, p = 0.006), with lower rates of AP users in regions with higher rates of mental health professionals (Fig 1).

A similar relationship was observed for mental health nurses (Kendall's tau -0.485, p = 0.002) but not for psychiatrists (Kendall's tau -0.181, p = 0.263), psychologists (Kendall's tau 0.228, p = 0.155) and educators (Kendall's tau -0.040, p = 0.833). After adjustment for possible confounders (Table 2), the total availability of mental health professionals, and the availability of nurses, but not of psychiatrists, psychologists and educators, significantly predicted the rate of individuals exposed to AP drugs (Table 2).

**Table 2:** Multivariate linear regression models investigating the association between mental health staff availability (total, mental health nurses, psychiatrists) and the rate of individuals prescribed antipsychotic drugs in Italy.

Independent variable	Model 1*		Model 2**	
	β coefficient (95% CI)	p-value	β coefficient (95% CI)	p-value
Total mental health staff	-0.266 (-0.497 to -0.034)	0.024	-0.296 (-0.622 to 0.029)	0.074
Mental health nurses	-0.792 (-1.431 to -0.153)	0.015	-0.956 (-1.770 to -0.142)	0.021
Psychiatrists	-1.317 (-2.978 to 0.343)	0.120	-1.562 (-14.768 to 11.644)	0.817
Psychologists	-0.356 (-2.857 to 2.145)	0.780	-1.253 (-47.940 to 45.434)	0.958
Educators / other staff	-0.001 (-0.062 to 0.062)	0.997	-0.006 (-0.098 to 0.085)	0.822

* Adjusted for the following confounding variables: psychiatric beds (x 100,000 inhabitants); treated prevalence of mental disorders (x 100,000 inhabitants); treated incidence of mental disorders (x 100,000 inhabitants), psychiatric hospital admissions (x 100,000 inhabitants); poverty index; employment rate.

** Adjusted for the following confounding variables: psychiatric beds (x 100,000 inhabitants); treated prevalence of schizophrenia (x 100,000 inhabitants); treated prevalence of bipolar disorder (x 100,000 inhabitants); treated incidence of schizophrenia (x 100,000 inhabitants); treated incidence of bipolar disorder (x 100,000 inhabitants); psychiatric hospital admissions (x 100,000 inhabitants); poverty index; employment rate.

## Discussion

Analysis of Italian national statistics found an inverse relationship between mental health staffing levels and AP prescribing. Intriguingly, when the analysis was carried out by type of staff, a statistical association was found for nurses but not for other professionals. We note, however, that the association coefficients for psychiatrists and psychologists were similar in magnitude to that for nurses, but with wider confidence intervals, therefore suggesting a similar general trend.

To our knowledge, this is the first study highlighting an association between staffing levels and AP prescribing in community mental healthcare. Interestingly, however, very similar results were found by a number of epidemiological studies carried out in nursing home settings, where staffing level was found to be inversely associated with AP use [6,7]. This association was particularly evident for nurses, who play a critical role in the caring of nursing home residents [6,7].

In community mental healthcare, however, the interpretation of this association is not straightforward, and the Italian data would suggest the following considerations. First, the rate of individuals exposed to AP medications is five times higher than the treated prevalence of schizophrenia and bipolar disorder considered together, and 1.5 higher than the treated prevalence of any mental disorder. Second, the rate of individuals prescribed AP medications is extremely variable in Italian regions, ranging from 1 to 4%. Third, one of the main challenges to studying AP utilization as a variable is determining appropriate versus inappropriate use. It would be incorrect, based on these aggregate statistics, to a priori imply that higher AP prescribing is negative. The existence of an inverse relationship does not guarantee inappropriate use as other factors may impact prescriber decisions to use APs. It remains therefore unclear what these data imply in terms of AP prescribing quality. For all these reasons, the possibility that increasing mental health staff may lead to better community mental health care and, consequently, less psychopathology and less AP use, remains a challenging hypothesis generated, but not addressed, by the present analysis. In any case, we demonstrated that a facility-level factor was associated with AP prescribing, which is a new key finding from a health system perspective.

This study has limitations. A first issue is that only data aggregated at regional level were accessed and analysed. This inevitably decreased statistical power, especially when multiple variables were included in the regression models. Additionally, information aggregated at regional level implies that within each Italian region service and AP prescribing data were homogeneous. We acknowledge that this assumption could not be verified and that the same analysis should be replicated at a local rather than regional level in order to increase statistical power, and to be based on fewer assumptions. A second limitation is that no outcome data were analysed to investigate the implications of different rates of AP prescribing at a population level. A third limitation is that although we adjusted for a number of potential confounders, we cannot exclude that residual confounding might have acted in an unpredictable way. For example, we could not adjust the analysis for disease severity. Finally, we cannot exclude that the rate of AP prescribing has been slightly overestimated as Italian regions collect prescribing data from two different sources. Additionally, as with all analyses of prescription databases, the lack of data on whether patients eventually took the prescribed agents should be highlighted, since a relevant proportion of the medicines prescribed for people with chronic conditions are not taken.

This study has practical implications. The finding that staffing level, a variable related to the organization of mental health systems, is associated with the observed variability in the rate of AP prescribing, suggests that any actions aimed at decreasing such variability need to take into account, in addition to patient- and prescriber-related factors, aspects related to the organization of the mental health system, in particular its staffing level. It is perhaps not surprising that mental health care exhibited a high degree of geographic variability. It has been shown that dissemination and adoption of treatments are influenced by many factors other than patient needs and scientific evidence, including marketing, profit motives, health care cultures, professional and guild issues, spending levels, organizational structures, financing mechanisms, and implementation difficulties [11,12]. In our analysis it is possible that spending levels influenced staffing level and organizational structures which, in turn, may have affected the provision of psychosocial interventions. Similarly, mental health care culture and professional issues may have affected AP prescribing as well as the provision of psychosocial interventions.

Unfortunately, the present study was not able to identify any potential mechanisms that may underlie the association between staffing levels and AP prescribing. It might be speculated that in regions with high staffing levels psychological, psychosocial and rehabilitation interventions were provided on a more regular basis, or more intensively or more appropriately, than in regions with low staffing level. As a consequence, individuals with mental disorders might have required less AP prescribing given the provision of non-pharmacological interventions. By contrast, in regions with low staffing levels, paucity of human resources might have implied limited provision of psychosocial and rehabilitation interventions, leaving AP prescribing as one of the few available therapeutic options.

In terms of implications for research, we argue that current evidence of correlation between staffing and AP prescribing warrants ad hoc studies to demonstrate potential causality.
